# Supplementary material for: Comparative Cytogenetic Abnormalities in Paired Choroidal Melanoma Samples Obtained Before and After Proton Beam Irradiation by Transscleral Fine-Needle Aspiration Biopsy and Endoresection
Source: Cancers (Basel). 2019 Aug 14;11(8):1173. doi: 10.3390/cancers11081173 (PMC6721816; doi:10.3390/cancers11081173)
Supplement: Supplementary file 1 [file cancers-11-01173-s001.pdf]

Supplementary Materials: Comparative Cytogenetic Abnormalities in Paired Choroidal Melanoma Samples Obtained Before and After Proton Beam Irradiation by Transscleral Fine-Needle Aspiration Biopsy and Endoresection

Alexandre Matet, Khadija Aït Raïs, Denis Malaise, Martina Angi, Rémi Dendale, Sarah Tick, Livia Lumbroso-Le Rouic, Christine Lévy-Gabriel, Manuel Rodrigues, Gaëlle Pierron and Nathalie Cassoux

Table S1. Detailed characteristics of 24 cases with paired choroidal melanoma samples from fine-needle aspiration biopsy and endoresection.

Case #	Age at diagnosis, year	Tumor largest basal diameter, mm	Tumor height, mm	Time from FNAB to proton therapy, month	Time from FNAB to EndoR, month	Disease-free survival, year	Meta-stastis, Yes/No	Death, Yes/No
1	66.4	13.0	12.3	0.5	3.4	5.42	No	No
2	60.8	9.6	9.6	0.5	2.1	1.58	Yes	Yes
3	52.3	12.3	8.8	0.6	2.3	0.75	No	No
4	27.8	12.5	9.7	0.6	3.1	3.58	No	No
5	64.5	13.4	9.7	0.5	2.0	3.33	No	No
6	36.7	11.3	4.8	0.5	3.5	2.92	No	No
7	47.6	8.5	8.1	0.5	2.0	3.67	No	No
8	34.8	12.8	10.7	0.6	1.9	0.67	No	No
9	40.4	15.0	10.4	0.7	2.2	5.75	No	No
10	49.8	14.9	8.7	0.6	2.1	3.33	No	No
11	63.8	14.2	8.3	0.5	2.2	2.75	No	No
12	73.9	15.7	10.6	0.6	2.4	2.50	Yes	No
13	54.0	8.3	9.6	0.6	2.3	4.17	No	No
14	42.7	10.0	8.4	0.5	2.0	5.17	No	No
15	33.5	9.7	7.5	0.6	1.9	4.33	No	No
16	52.8	12.8	5.1	0.6	3.0	5.42	No	No
17	48.3	9.9	8.3	0.5	2.5	0.92	No	No
18	66.5	13.8	8.7	0.5	2.2	4.08	No	No
19	20.8	15.3	8.4	0.5	2.8	5.67	No	No
20	61.8	13.2	7.5	0.5	2.0	4.33	No	No
21	59.5	14.8	7.4	0.5	2.3	5.30	Yes	No
22	40.3	15.0	9.5	0.6	3.5	5.58	No	No
23	42.3	9.2	8.9	0.7	2.5	2.33	No	No
24	62.7	12.5	6.2	0.6	2.1	4.00	No	No

FNAB= fine-needle aspiration biopsy; EndoR= endoresection; #= number.

© 2019 by the authors. Licensee MDPI, Basel, Switzerland. This article is an open access article distributed under the terms and conditions of the Creative Commons Attribution (CC BY) license (http://creativecommons.org/licenses/by/4.0/).
